# Polyethylene Glycol-Assisted Engineering of NiCo_2_S_4_ Nanostructures for Enhanced Supercapacitor Performance

**DOI:** 10.3390/polym18091026

**Published:** 2026-04-24

**Authors:** Pritam J. Morankar, Aviraj M. Teli, Sonali A. Beknalkar, Chan-Wook Jeon

**Affiliations:** 1 School of Chemical Engineering, Yeungnam University, 280 Daehak-ro, Gyeongsan 38541, Republic of Korea; pritam.nanoworld@gmail.com; 2 Division of Electronics and Electrical Engineering, Dongguk University, Seoul 04620, Republic of Korea

**Keywords:** NiCo_2_S_4_, PEG-assisted synthesis, hydrothermal method, nanostructured electrode, asymmetric supercapacitor, areal capacitance, energy density, cycling stability

## Abstract

The development of high-performance electrode materials with controlled morphology remains a key challenge for advancing supercapacitor technologies. In this study, polyethylene glycol (PEG)-assisted hydrothermal synthesis was employed to engineer NiCo_2_S_4_ nanostructures with controlled morphology for enhanced supercapacitor performance. The influence of PEG concentration on nucleation behavior, structural evolution, and electrochemical characteristics was systematically investigated. The optimized NiCo_2_S_4_ electrode synthesized with 0.2% PEG (NiCoS-P2) exhibited a hierarchical flower-like nanosheet architecture with reduced agglomeration and improved electrochemically accessible surface area. As a result, the electrode delivered a high areal capacitance of 13.689 F/cm^2^ (specific capacitance of 6845 F/g) at 5 mA/cm^2^, along with excellent rate capability and superior cycling stability, retaining 84.16% capacitance after 12,000 cycles. Electrochemical analysis revealed that the charge storage process is predominantly diffusion-controlled with enhanced ion transport kinetics. Furthermore, an asymmetric supercapacitor device assembled using NiCoS-P2 as the positive electrode and activated carbon as the negative electrode demonstrated a wide operating voltage of 1.5 V, delivering an areal capacitance of 0.409 F/cm^2^ (specific capacitance of 204.5 F/g), an energy density of 0.128 mWh/cm^2^, and a power density of 2.99 mW/cm^2^. The device also exhibited excellent long-term stability with 85.3% capacitance retention after 7000 cycles. This work highlights the effectiveness of polymer-assisted structural engineering in optimizing transition metal sulfide electrodes for advanced energy storage applications.:

## 1. Introduction

The ever-increasing environmental consequences associated with excessive fossil fuel consumption, coupled with the progressive depletion of conventional energy resources, have intensified global efforts toward the development of sustainable and efficient energy technologies [1,2]. As nations increasingly transition toward renewable energy systems and electrified infrastructures, the demand for advanced energy storage devices capable of accommodating fluctuating energy supply and rapidly varying power requirements has grown substantially [3]. In particular, the widespread integration of renewable energy sources such as solar and wind into modern power networks necessitates energy storage technologies that can ensure reliable energy management, rapid charge–discharge capability, and long-term operational stability [4]. Consequently, the development of high-performance electrochemical energy storage systems has emerged as a critical research priority for supporting next-generation energy infrastructures [5]. Among the various electrochemical energy storage technologies, supercapacitors have attracted considerable attention owing to their high power density, rapid charge–discharge capability, and outstanding cycling durability [6]. Unlike conventional batteries that rely primarily on diffusion-controlled electrochemical reactions within the bulk of electrode materials, supercapacitors store energy through electric double-layer and Faradaic (battery-type Faradaic or pseudocapacitive) processes depending on electrode material, enabling much faster energy delivery and superior cycle life. These distinctive characteristics make supercapacitors highly suitable for applications requiring rapid energy supply, such as hybrid electric vehicles, regenerative braking systems, backup power sources, and portable electronic devices [7]. Despite these advantages, the relatively low energy density of supercapacitors compared with battery systems remains a major limitation that restricts their broader implementation in high-energy storage applications [8].

The electrochemical performance of supercapacitors is largely governed by the nature of the electrode materials employed. Carbon-based materials such as activated carbon, graphene, and carbon nanotubes store charge mainly through electric double-layer capacitance and generally exhibit excellent electrical conductivity and long-term stability [9]. However, the energy storage capability of these materials is relatively limited because the charge storage process is primarily confined to physical adsorption at the electrode–electrolyte interface [10]. To overcome this limitation, battery-type Faradaic materials based on transition metal compounds have been extensively investigated due to their ability to store charge through fast and reversible faradaic redox reactions [11]. Transition metal oxides and hydroxides, including NiO, Co_3_O_4_, and MnO_2_, have demonstrated promising electrochemical properties owing to their high theoretical capacitance and multiple oxidation states. In addition, emerging materials such as transition metal phosphides, nitrides, and MXene-based systems have attracted significant attention due to their enhanced electrical conductivity and rapid redox kinetics [12]. Nevertheless, these materials often suffer from intrinsic drawbacks such as poor electrical conductivity, restricted ion diffusion, and structural degradation during repeated charge–discharge processes [13]. In recent years, transition metal sulfides have emerged as attractive alternatives to oxide-based electrode materials because of their superior electrical conductivity and enhanced electrochemical activity [14]. The lower electronegativity of sulfur compared with oxygen facilitates more efficient electron transport and promotes favorable redox kinetics during electrochemical reactions [15]. Consequently, various sulfide-based materials, including NiS, CoS, and CuCo_2_S_4_, have been explored as promising electrode candidates for high-performance supercapacitor applications based on battery-type Faradaic charge storage behavior. Among them, nickel cobalt sulfide (NiCo_2_S_4_) has received considerable attention due to its spinel crystal structure and the presence of multiple valence states associated with nickel and cobalt ions. These characteristics provide abundant electrochemically active sites and facilitate rapid charge transfer, which are essential for achieving high electrochemical performance [16]. Despite these advantages, the practical electrochemical performance of NiCo_2_S_4_ electrodes is often hindered by several structural limitations, including particle agglomeration, insufficient electrochemically active surface area, and sluggish ion diffusion within densely packed structures [17]. These issues reduce the effective utilization of active materials and negatively influence both rate capability and cycling stability. Therefore, controlling the morphology and nanostructure of NiCo_2_S_4_ materials has become an important strategy for enhancing their electrochemical performance [18].

Several studies have reported various approaches to improve the structural and electrochemical properties of NiCo_2_S_4_-based electrodes. For example, Ramesh et al. synthesized NiCo_2_S_4_@N-MWCNT and NiCo_2_S_4_@N-MWCNT/MOF-67 composites through a sonication-assisted hydrothermal method, where the MOF-derived composite exhibited enhanced capacitance of approximately 455 F g^−1^ at 1 A g^−1^ with excellent capacitance retention in alkaline electrolyte [19]. Shinde et al. prepared NiCo_2_S_4_ nanostructured electrodes using the successive ionic layer adsorption and reaction technique and reported a capacitance of 1076 F g^−1^ at optimized deposition cycles [20]. Chen et al. further demonstrated caterpillar-like NiCo_2_S_4_ nanosheets decorated with nanowires grown on nickel foam, delivering a high specific capacitance of 1777 F g^−1^ at 1 A g^−1^ along with good cycling stability [21]. Similarly, Shi et al. developed a hierarchical NiCo_2_S_4_@Ni(OH)_2_ core–shell architecture on carbon cloth, achieving a high specific capacity of 404.2 mAh g^−1^ and an energy density of 83 Wh kg^−1^ in an assembled asymmetric supercapacitor device [22]. Apart from structural engineering strategies, polymer-assisted synthesis has also been considered an effective approach to regulate the nucleation and growth behavior of nanostructured materials [23]. For instance, Aziz et al. fabricated P4VPy/ NiCo_2_S_4_/PANI nanocomposites and observed a substantial enhancement in capacitance compared with pristine P4VPy due to the synergistic interaction between the conductive polymer and the metal sulfide framework [24]. Similarly, Sami et al. prepared NiCoS-coated polyacrylonitrile nanofibers through electrospinning followed by electrodeposition, achieving high capacitance and improved rate capability owing to the conductive fibrous architecture that facilitated efficient electron transport [25]. Among the various polymeric additives used during nanomaterial synthesis, PEG has been widely employed because of its good solubility and ability to influence nucleation and crystal growth processes [26]. PEG molecules can provide steric stabilization during particle formation, which helps reduce aggregation and promotes the formation of more uniformly distributed nanostructures. Moreover, variations in the molecular weight of PEG can influence the growth environment of nanomaterials and consequently affect their morphology and electrochemical properties [27].

In this study, a polymer-assisted hydrothermal strategy was employed to synthesize NiCo_2_S_4_ nanostructures using PEG as a soft growth regulator. The effect of PEG concentration on nucleation behavior, structural evolution, and morphological characteristics was systematically investigated. The optimized electrode exhibits a hierarchical nanosheet-based architecture with reduced agglomeration and enhanced electrochemically accessible surface area, resulting in improved electrochemical performance, including higher capacitance, better rate capability, and stable cycling behavior. Beyond morphological control, this work further examines the influence of PEG on electrochemical kinetics. By correlating PEG concentration with ion transport behavior, the charge storage mechanism is analyzed using diffusion coefficient estimation, b-value evaluation, and separation of capacitive and diffusion-controlled contributions. The results suggest that the optimized structure facilitates more efficient ion diffusion and improved reaction kinetics. This provides a clearer understanding of the relationship between structure and electrochemical performance, offering additional insight beyond conventional morphology-focused studies.

## 2. Materials and Methods

### 2.1. Synthesis of PEG-Assisted NiCo_2_S_4_ Electrodes

**Figure 1:**
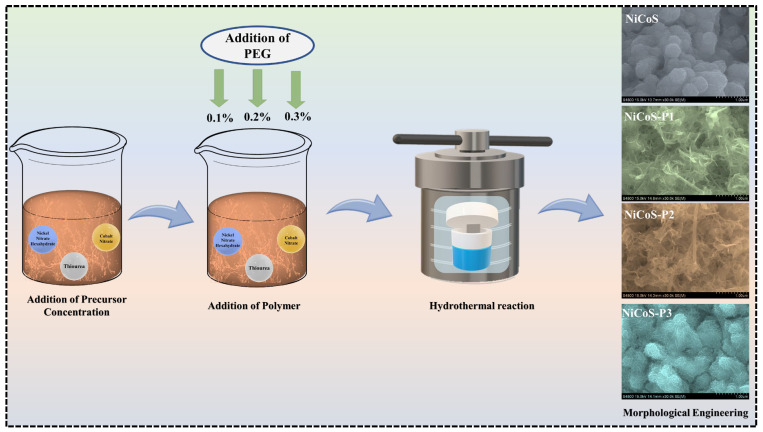
Schematic illustration of the synthesis procedure for pristine NiCoS and PEG-assisted NiCo_2_S_4_ electrodes.

NiCo_2_S_4_ electrodes were synthesized on nickel foam via a hydrothermal method using PEG (A.M.W.—20,000 g/mol, Sigma-Aldrich U.S., St. Louis, MO, USA) as a polymeric growth regulator. In a typical process, 0.05 M of nickel nitrate hexahydrate (Ni (NO_3_)_2_·6H_2_O, Sigma-Aldrich U.S.) and 0.1 M of cobalt nitrate hexahydrate (Co (NO_3_)_2_·6H_2_O, Sigma-Aldrich U.S.) were dissolved in 100 mL of deionized (DI) water under continuous stirring. Subsequently, 0.2 M of thiourea (NH_2_CSNH_2,_ Sigma-Aldrich U.S.) was added as the sulfur source, and the mixture was stirred for 20 min to obtain a homogeneous precursor solution. PEG was then introduced at concentrations of 0.1%, 0.2%, and 0.3%, followed by stirring for 30 min to ensure uniform dispersion of the polymer within the solution. Nickel foam (2 × 3 cm^2^) was cleaned by ultrasonication in 3 M HCl, ethanol, and DI water for 10 min each to remove surface impurities. The cleaned substrate was immersed in the prepared precursor solution and transferred into a Teflon-lined stainless-steel autoclave, which was maintained at 180 °C for 12 h. During the hydrothermal process, PEG molecules regulated the nucleation and growth of NiCo_2_S_4_, leading to improved structural uniformity and reduced particle aggregation. After naturally cooling to room temperature, the coated nickel foam was removed, thoroughly washed with DI water and ethanol, and dried at 80 °C overnight. The obtained electrodes were further annealed at 400 °C for 2 h in air to enhance crystallinity and structural stability. The mass loading of the active material on the nickel foam was approximately 2 mg cm^−2^, determined from the weight difference before and after deposition. For comparison, the sample synthesized without PEG was denoted as NiCo_2_S_4_ (NiCoS), while the PEG-assisted samples were labeled as NiCoS-P1, NiCoS-P2, and NiCoS-P3, corresponding to 0.1%, 0.2%, and 0.3% PEG, respectively. A schematic illustration of the PEG-assisted synthesis process is shown in Figure 1.

### 2.2. Sample Characterization and Electrochemical Measurements

The structural properties of the prepared NiCoS and PEG-assisted NiCoS-P1, NiCoS-P2, and NiCoS-P3 electrodes were analyzed using X-ray diffraction (XRD) with Cu Kα radiation (λ = 1.5406 Å) to determine their phase composition and crystallinity. The surface morphology and microstructural features were examined through field-emission scanning electron microscopy (FE-SEM) (S4800, HITACHI, Tokyo, Japan). In addition, energy-dispersive X-ray spectroscopy (EDS) attached to the FESEM instrument(Hitachi S-4800, Chiyoda, Tokyo, Japan) was used to verify the elemental composition and distribution within the synthesized materials. The surface chemical states and oxidation states of the constituent elements were further investigated using X-ray photoelectron spectroscopy (XPS) (K-Alpha, Thermo Scientific, Cheshire, UK). All XPS spectra were calibrated using the instrument’s built-in charge compensation and internal calibration system; no external C 1s reference peak was used. Electrochemical measurements were performed using a Biologic WBCS3000 electrochemical workstation (BioLogic Science Instruments, Seyssinet-Pariset, France) in a standard three-electrode configuration. The synthesized electrode served as the working electrode, while a platinum wire and an Ag/AgCl electrode were used as the counter and reference electrodes, respectively. All electrochemical tests were carried out in 2 M KOH aqueous electrolyte to evaluate the capacitive behavior, charge–discharge performance, and cycling stability of the electrodes.

## 3. Results and Discussions

### 3.1. XRD Elucidation

**Figure 2:**
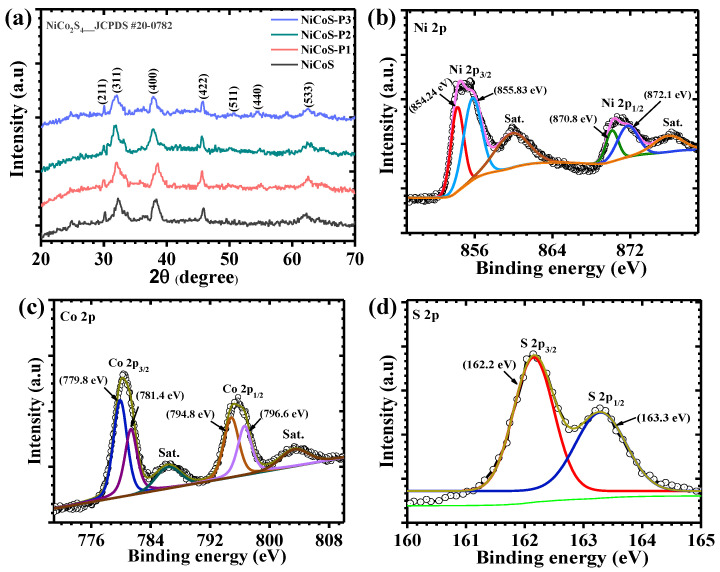
(**a**) XRD patterns of all studied electrodes, High-resolution XPS spectra of the optimized NiCoS-P2 electrode, displaying (**b**) Ni 2p, (**c**) Co 2p, and (**d**) S 2p regions.

The XRD patterns of NiCoS, NiCoS-P1, NiCoS-P2, and NiCoS-P3 are presented in Figure 2a. All diffraction peaks can be indexed to the cubic spinel phase of NiCo_2_S_4_, which agrees well with the standard JCPDS card No. 20-0782, confirming the successful formation of the desired material. The prominent diffraction peaks observed at 2θ ≈ 29.9°, 31.9°, 37.9°, 46.6°, 50.3°, 55.1°, and 61.9° correspond to the (211), (311), (400), (422), (511), (440), and (533) crystallographic planes of NiCoS, respectively [28]. No additional peaks related to impurity phases are detected, indicating the high phase purity of the synthesized samples. The peak positions remain nearly unchanged after the introduction of PEG, suggesting that the polymer does not alter the fundamental crystal structure of NiCoS. However, a slight increase in peak intensity and improved peak definition can be observed in the PEG-assisted samples, indicating a marginal improvement in crystallinity. This behavior suggests that PEG influences the nucleation and growth of NiCoS during the hydrothermal process, leading to more uniform crystallite formation. Such improved crystallinity may facilitate efficient electron transport within the material, which is beneficial for electrochemical charge storage applications [29].

### 3.2. XPS Analysis

To examine the surface chemical states and elemental composition, XPS was performed for the optimized NiCoS-P2 electrode, and the corresponding spectra are presented in Figure 2b–d. The analysis confirms the presence of Ni, Co, and S elements, verifying the successful formation of the Ni–Co sulfide structure [30]. The high-resolution Ni 2p spectrum (Figure 2b) shows two main spin–orbit peaks located at approximately 854.2 eV and 872.1 eV, corresponding to Ni 2p_3/2_ and Ni 2p_1/2_, respectively. Additional peaks observed at around 855.3 eV and 870.8 eV are associated with higher valence nickel species [31]. The presence of distinct satellite peaks at higher binding energies further confirms the characteristic electronic structure of nickel in sulfide-based materials. The coexistence of multiple nickel oxidation states indicates the presence of active redox sites that can contribute to electrochemical reactions. The Co 2p spectrum (Figure 2c) exhibits two dominant peaks at approximately 779.8 eV and 794.8 eV, which correspond to Co 2p_3/2_ and Co 2p_1/2_, respectively. The peaks located at 781.4 eV and 796.6 eV can be attributed to oxidized cobalt species, while the additional features at higher binding energies are assigned to satellite peaks [32]. These features are typical for cobalt-containing sulfide compounds and indicate the presence of mixed cobalt oxidation states within the structure. The S 2p spectrum (Figure 2d) displays characteristic peaks corresponding to sulfur species in metal sulfides. The peaks appearing at approximately 162.2 and 163.3 eV can be attributed to S 2p_3/2_ and S 2p_1/2_, which are associated with the metal–sulfur (M–S) bonding in the NiCoS structure. The presence of these peaks confirms the successful incorporation of sulfur into the metal framework and the formation of the Ni–Co sulfide phase. The XPS results verify the presence of Ni, Co, and S elements in the synthesized material, confirming the formation of the Ni–Co sulfide framework. The coexistence of multiple oxidation states for nickel and cobalt suggests a rich redox chemistry within the structure. Such electronic features can promote efficient charge transfer and provide abundant electrochemically active sites, which are expected to enhance the overall electrochemical behavior of the NiCoS-P2 electrode [33].

### 3.3. Morphological and Elemental Composition

**Figure 3:**
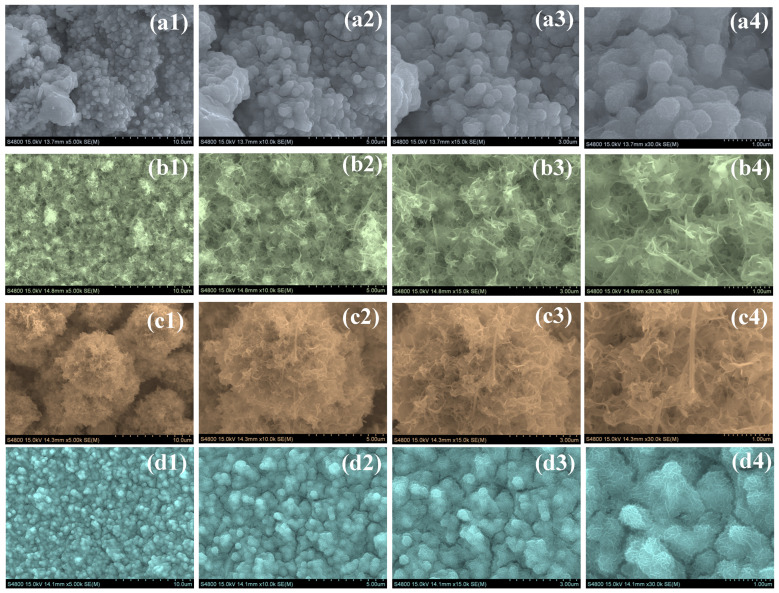
FESEM micrographs of (**a1**–**a4**) pristine NiCoS, (**b1**–**b4**) NiCoS-P1, (**c1**–**c4**) NiCoS-P2, and (**d1**–**d4**) NiCoS-P3.

The surface morphology of the synthesized NiCoS, NiCoS-P1, NiCoS-P2, and NiCoS-P3 electrodes was examined by FESEM, and the corresponding images at different magnifications are presented in Figure 3(a1–d4). The NiCoS sample depicted in Figure 3(a1–a4) shows a surface composed of closely packed nanograins that form irregular agglomerated clusters. These particles are interconnected and distributed over the substrate, producing a relatively compact morphology. At higher magnification, the structure appears as aggregated granular domains formed through the assembly of primary nanoparticles. For the NiCoS-P1 sample Figure 3(b1–b4), the introduction of PEG leads to noticeable changes in the surface structure. The morphology evolves into a more open arrangement consisting of thin nanosheet-like structures. These nanosheets are randomly oriented and partially overlapped, creating an interconnected framework with visible pores between the sheets. The NiCoS-P2 sample Figure 3(c1–c4) exhibits the formation of well-developed hierarchical structures. The surface is mainly composed of flower-like assemblies constructed from ultrathin nanosheets. These nanosheets are loosely stacked and radially arranged, forming a porous three-dimensional network. The open framework provides increased surface exposure and creates pathways that can facilitate electrolyte penetration and ion transport. In contrast, the NiCoS-P3 sample Figure 3(d1–d4) displays densely packed nanostructured clusters. The nanograins appear closely assembled, producing a relatively compact structure compared with the nanosheet-based architecture observed for NiCoS-P2. The morphological evolution observed across the samples suggests that PEG influences the nucleation and growth behavior of NiCoS during the hydrothermal synthesis. The interaction between PEG and the precursor species can regulate crystal growth and particle assembly, leading to variations in structural organization. The interconnected nanosheet framework observed in NiCoS-P2 provides a porous architecture that can enhance electrolyte accessibility and support efficient charge transport during electrochemical processes.

**Figure 4:**
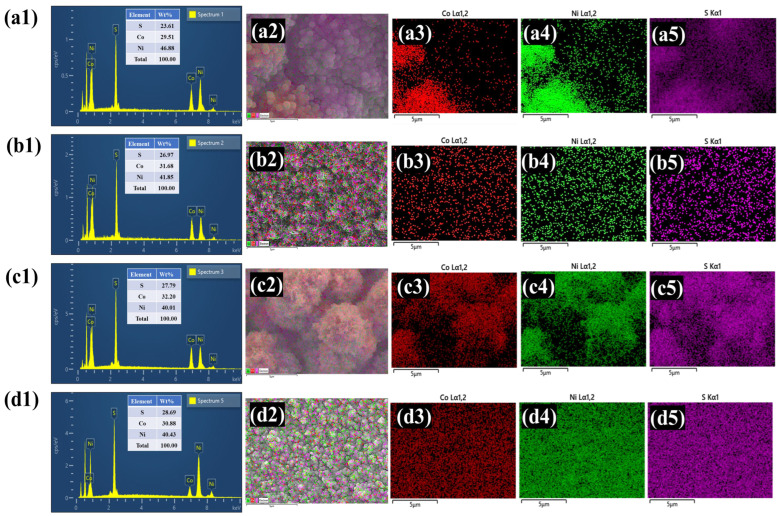
EDS spectra along with corresponding elemental mapping images of (**a1**–**a5**) pristine NiCoS, (**b1**–**b5**) NiCoS-P1, (**c1**–**c5**) NiCoS-P2, and (**d1**–**d5**) NiCoS-P3.

The elemental composition of the prepared NiCoS, NiCoS-P1, NiCoS-P2, and NiCoS-P3 electrodes was analyzed using EDS coupled with the FESEM instrument. The EDS spectra presented in Figure 4(a1–d1) exhibit distinct peaks corresponding to Ni, Co, and S, confirming the successful formation of the nickel–cobalt sulfide phase. The elemental weight percentages shown in the inset further support the presence of these elements in ratios consistent with the expected composition of the synthesized material. To examine the spatial distribution of the elements, EDS elemental mapping was carried out for the representative electrode. As shown in Figure 4(a2–d5), the mapping images reveal that nickel, cobalt, and sulfur are evenly distributed across the entire electrode surface, with no evidence of elemental clustering or segregation. This uniform dispersion indicates that the synthesis process enables consistent nucleation and growth of the NiCoS structure. Such a homogeneous elemental arrangement is beneficial for maintaining structural stability and facilitating efficient charge transfer during electrochemical operation.

## 4. Electrochemical Analysis

**Figure 5:**
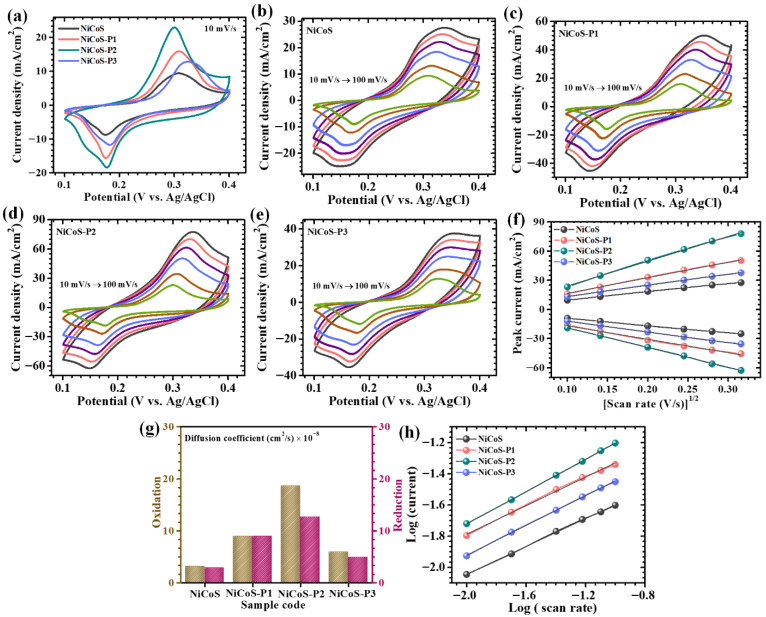
CV analysis of pristine and PEG-modified electrodes: (**a**) CV curves recorded at 10 mV/s, (**b**–**e**) CV profiles at varying scan rates (10–100 mV/s), (**f**) Relationship between peak current and square root of scan rate, (**g**) Comparison of diffusion coefficients, and (**h**) Determination of b-values.

The electrochemical performance of the synthesized NiCo_2_S_4_ electrodes, with systematically varied polyethylene glycol (PEG) concentrations (0, 0.1, 0.2, and 0.3%), was comprehensively investigated to elucidate the role of polymer-assisted structural modulation on charge storage characteristics. The incorporation of PEG is expected to influence nucleation kinetics, particle dispersion, and hierarchical assembly, thereby directly impacting electrochemical behavior. CV measurements recorded at scan rate of 10 mV/s, reveal that all electrodes exhibit well-defined anodic and cathodic peaks within the selected potential window (0.1 to 0.4 V versus Ag/AgCl), confirming that the charge storage mechanism is predominantly governed by battery-type Faradaic behavior (Figure 5a). These redox features arise from reversible transitions of Ni^2+^/Ni^3+^ and Co^2+^/Co^3+^ species, which actively participate in electrochemical reactions through interaction with hydroxide ions in the electrolyte [34]. A distinct dependence of electrochemical response on PEG concentration is observed. The pristine NiCoS electrode displays relatively broad peaks with lower current intensity, indicating limited electroactive surface exposure and slower reaction kinetics, primarily due to particle aggregation during synthesis. Upon introducing PEG, a noticeable improvement in electrochemical response is achieved. The NiCoS-P1 electrode shows enhanced peak definition and increased current response, suggesting improved dispersion of active material and partial suppression of agglomeration. The electrode with 0.2% PEG (NiCoS-P2) exhibits the most pronounced redox peaks and the largest CV area, indicating optimal electrochemical activity. This enhancement can be attributed to the role of PEG as a soft structure-directing agent. The polymer chains introduce steric hindrance during nucleation and growth, leading to uniform particle distribution and the formation of a porous, interconnected architecture. Such structural features significantly improve electrolyte accessibility and facilitate rapid ion diffusion [35]. In contrast, further increasing the PEG concentration to 0.3% (NiCoS-P3) results in a decline in electrochemical performance. Excessive PEG increases solution viscosity and can lead to over-coating or partial surface blockage, thereby hindering electron transport and restricting access to active sites. The near-symmetric nature of redox peaks across all samples indicates quasi-reversible electrochemical behavior. The optimized NiCoS-P2 electrode demonstrates the highest degree of symmetry, suggesting reduced polarization and improved reaction reversibility. The influence of scan rate on electrochemical behavior was systematically evaluated by varying scan rate from 10 to 100 mV/s, shown in Figure 5b–e. With increasing scan rate, all electrodes exhibit a proportional increase in current response, while maintaining the general shape of CV curves. This indicates stable redox activity and good electrochemical reversibility. At higher scan rates, a slight shift in peak potentials is observed due to polarization effects [36]. However, the shift is less pronounced for the optimized electrode, indicating faster electron transfer kinetics and lower internal resistance. The ability to preserve the redox profile at high scan rates highlights the superior rate capability of the NiCoS-P2 electrode.

To further elucidate the ion transport dynamics and redox kinetics, the CV responses recorded at different scan rates (10–100 mV/s) were systematically analyzed. As illustrated in Figure 5f, a linear relationship between the peak current (*i_p_*) and the square root of the scan rate (*v*^1/2^) is observed for all electrodes, indicating that the electrochemical processes are predominantly governed by battery-type Faradaic reactions with diffusion-controlled Faradaic reactions. To quantitatively evaluate the ion diffusion characteristics, the apparent diffusion coefficients (D) of the bare and PEG-modified NiCo_2_S_4_ electrodes were determined using the Randles–Sevcik Equation (1) [37]:

(1) $$D=\frac{i_{p}}{2.69\;\times \;10^{5\;}\times \;n^{3/2\;}\times \;A\;\times \;C\;\times \;v^{1/2}}$$

where *i_p_* represents the peak current, *n* is the number of electrons involved in the redox process, *A* denotes the electrochemically active surface area, *C* is the electrolyte ion concentration, and *v* is the scan rate. The calculated diffusion coefficients at a representative scan rate (10 mV/s) are summarized in Table 1, with their comparative distribution depicted in Figure 5g. A clear trend emerges, wherein the NiCoS-P2 electrode exhibits the highest diffusion coefficient for both anodic and cathodic processes, highlighting its superior ion transport capability and accelerated reaction kinetics. In contrast, the pristine NiCoS electrode demonstrates comparatively lower diffusion coefficients, primarily due to particle aggregation and limited accessible surface area, which impede effective ion transport. Similarly, the NiCoS-P1 sample, although showing some improvement, suffers from incomplete morphological evolution due to insufficient polymer content, resulting in suboptimal diffusion pathways. On the other hand, excessive PEG incorporation (NiCoS-P3) leads to increased viscosity during synthesis and possible surface passivation effects, which hinder electrolyte accessibility and restrict ion mobility [38]. The observed variation in diffusion coefficients across the electrode series clearly underscores the critical influence of PEG concentration on microstructural evolution and electrochemical kinetics.

Further, to gain quantitative insight into the charge storage mechanism, the relationship between peak current and scan rate was analyzed using the power-law Equation (2) [39]:

(2) $$i={av}^{b}$$

where the exponent b provides critical insight into the governing charge storage mechanism. Typically, b values approaching 0.5 indicate diffusion-controlled Faradaic processes, whereas values closer to 1 are characteristic of surface-dominated capacitive behavior [39]. The b-values were extracted from the slope of the linear fitting of *log*(*i*) versus *log*(*v*) plots (Figure 5h), and the obtained values for all electrodes are summarized in Table 1. The extracted b-values fall within the range of approximately 0.44 to 0.51 for all studied samples. These results clearly demonstrate that the charge storage process is predominantly governed by battery-type Faradaic behavior, arising from diffusion-controlled redox reactions associated with ion intercalation and bulk transformations within the electrode matrix. The slightly lower b-value of 0.44 observed for the bare electrode compared to the ideal diffusion-controlled value (0.5) can be attributed to non-ideal behavior, such as limited ionic accessibility, higher internal resistance, and structural heterogeneity, which hinder efficient ion diffusion and charge transfer. At the same time, the slight deviation of b-values toward higher values indicates the presence of a measurable surface-controlled contribution, which becomes increasingly relevant due to improved electrode-electrolyte interaction [40].

To quantitatively distinguish the relative contributions of surface-controlled capacitive processes and diffusion-governed Faradaic reactions, the total current response was deconvoluted using the well-established relationship (3) [41]:

(3) $$i\left(V\right)=k_{1}v{+k_{2}v}^{1/2}$$

where the term *k*_1_*v* corresponds to the surface-controlled capacitive contribution, primarily associated with rapid charge accumulation at the electrode/electrolyte interface, while *k*_2_*v*^1/2^ represents the diffusion-controlled component arising from ion insertion and bulk redox reactions. The constants *k*_1_ and *k*_2_ were determined through linear fitting by plotting *i(V)/v*^1/2^ as a function of *v*^1/2^, enabling a reliable separation of the two charge storage mechanisms. Based on this analysis, the total stored charge (*Q_t_*) within the CV profiles can be expressed as the sum of capacitive (*Q_s_*) and diffusion-controlled (*Q_d_*) contributions (4) [41]:

(4) $$Q_{t}=Q_{s}+Q_{d}$$

**Figure 6:**
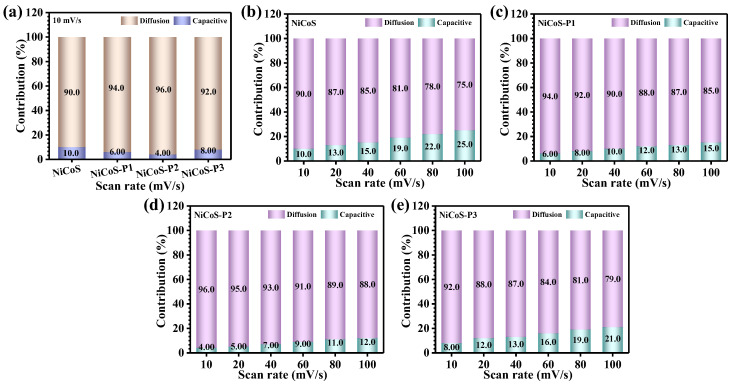
Charge storage kinetics analysis: (**a**) Separation of capacitive and diffusion-controlled contributions at 10 mV/s, and (**b**–**e**) quantitative contribution analysis for NiCoS and PEG-assisted NiCo_2_S_4_ electrodes at different scan rates.

The quantitative evaluation performed at a low scan rate (10 mV/s) reveals that diffusion-controlled processes dominate the overall charge storage for all electrodes. Notably, the NiCoS-P2 electrode exhibits the highest diffusion contribution (96%) (Figure 6a), clearly indicating that bulk Faradaic reactions are the primary charge storage mechanism in this system. This pronounced diffusion dominance reflects enhanced ion transport kinetics and efficient utilization of the electroactive material. The superior diffusion contribution observed for the optimized electrode can be directly correlated with its PEG-mediated structural features. As a result, a larger fraction of the electrode volume actively participates in redox reactions, leading to enhanced charge storage efficiency. Furthermore, the evolution of charge storage behavior with scan rate was systematically examined, shown in Figure 6b–e. As the scan rate increases, a gradual rise in the capacitive contribution is observed for all electrodes. This trend arises from the limited time available for electrolyte ions to penetrate deep into the electrode at higher scan rates, resulting in a shift toward surface-dominated charge storage. Consequently, fast surface redox reactions become increasingly prominent under these conditions [42]. Despite this shift, the NiCoS-P2 electrode consistently maintains a higher diffusion contribution across the entire scan rate range, underscoring its superior ion transport capability and structural optimization. This balanced combination of diffusion-controlled and capacitive processes enables efficient charge storage under both low and high-rate conditions.

**Figure 7:**
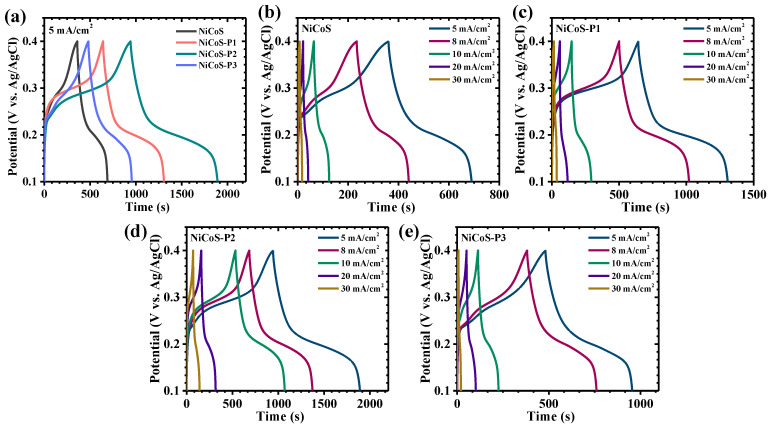
GCD characteristics of NiCoS and PEG-assisted electrodes: (**a**) GCD curves at 5 mA/cm^2^ and (**b**–**e**) Profiles at various current densities.

**Figure 8:**
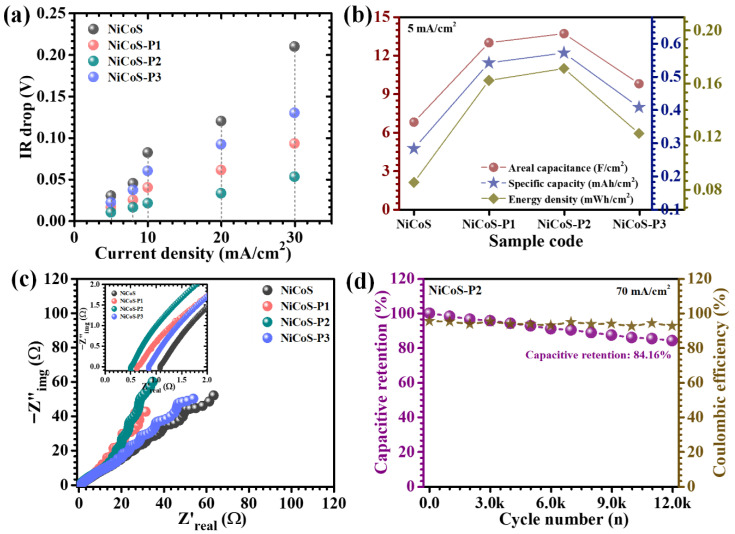
Comparative electrochemical performance of electrodes, including (**a**) IR drop, (**b**) energy storage parameters, (**c**) Nyquist plots, and (**d**) cycling stability of the NiCoS-P2 electrode up to 12,000 cycles.

The comparative GCD profiles recorded at a current density of 5 mA/cm^2^ clearly reveal distinct performance variations among the pristine and PEG-modified NiCo_2_S_4_ electrodes (Figure 7a). All samples exhibit non-linear charge–discharge behavior with well-defined potential plateaus, which is characteristic of battery-type Faradaic charge storage governed by reversible redox reactions rather than ideal electric double-layer capacitance. GCD measurements were furthermore systematically conducted at varying current densities from 5 to 30 mA/cm^2^ (Figure 7b–e). Among the investigated electrodes, the NiCoS-P2 sample demonstrates a markedly extended discharge duration compared to the pristine, NiCoS-P1, and NiCoS-P3 counterparts, indicating its superior charge storage capability. The prolonged discharge time directly reflects enhanced utilization of electroactive sites and improved redox efficiency. Furthermore, the discharge curves of the optimized electrode display a more gradual and smooth potential decay, signifying stabilized redox transitions and reduced polarization effects during operation [21]. Across all electrodes, the charge–discharge curves exhibit a high degree of symmetry between the charging and discharging branches, indicating excellent coulombic efficiency and reversible electrochemical behavior. A critical parameter reflecting internal resistance is the IR drop observed at the beginning of the discharge process. The optimized electrode exhibits the smallest IR drop among all samples, highlighting its reduced internal resistance and superior electrical conductivity (Figure 8a). The variation in IR drop with increasing current density further confirms this behavior, where higher current densities lead to increased voltage drop due to greater polarization [43].

To quantitatively assess the electrochemical performance, key parameters such as areal capacitance (C_A_), energy density (ED), and power density (PD) were calculated based on the GCD curves. In addition to areal capacitance, gravimetric capacitance (Cg, F/g) was calculated using the active material loading of 2 mg cm^−2^ to enable fair comparison with previously reported electrode materials. Considering the non-linear nature of the discharge profiles, the capacitance values were determined using an integrated approach to accurately capture the contribution of Faradaic processes (5)–(7) [44,45]:

(5) $$C_{A}=\frac{I\;\times \;2\;\times \;\int V\left(t\right)dt}{A\;\times {\;(∆V)}^{2}}$$

(6) $$ED=\frac{1}{2\times 3600}\;C_{A}\times {dV}^{2}$$

(7) $$PD=\frac{ED\times 3600}{T_{d}}$$

where *I* represents the discharge current, *∫V(t)dt* corresponds to the integrated area under the discharge curve, *A* is the electrode area, Δ*V* is the operating potential window, and *T_d_* denotes the discharge time. This methodology ensures accurate evaluation of charge storage performance for systems dominated by Faradaic reactions. The calculated areal and gravimetric capacitance values clearly indicate a strong dependence on PEG concentration (Table 2). The NiCoS-P2 electrode delivers the highest capacitance of 13.689 F/cm^2^ (6845 F/g) at 5 mA/cm^2^ current density, significantly outperforming the pristine and other PEG-modified electrodes (Figure 8b). This superior performance arises from the synergistic combination of increased electroactive surface area, improved electrical conductivity, and enhanced ion transport pathways. The rate capability of the electrodes was further evaluated by analyzing capacitance retention at increasing current densities. As expected, all samples exhibit a gradual decrease in capacitance with increasing current density, primarily due to limited ion diffusion into the deeper regions of the electrode at higher charge–discharge rates. However, the NiCoS-P2 electrode demonstrates significantly improved retention approximately 86.04% at 10 mA/cm^2^, maintaining a substantial fraction of its initial capacitance even at high current densities. This superior rate performance highlights the effectiveness of PEG-assisted structural engineering in enabling rapid ion and electron transport under demanding operating conditions.

EIS was employed to gain deeper insight into the charge transfer characteristics and ion diffusion behavior of the studied electrodes in alkaline electrolyte. The Nyquist plots (Figure 8c) exhibit the typical impedance features consisting of a high-frequency intercept on the real axis, a semicircular arc in the intermediate frequency region, and an inclined linear segment at low frequencies. The intercept at the high-frequency region corresponds to the equivalent series resistance (ESR), which includes contributions from the intrinsic resistance of the active material, electrolyte resistance, and contact resistance at the electrode–current collector interface. The diameter of the semicircle represents the charge transfer resistance (Rct), which reflects the kinetics of battery-type Faradaic reactions occurring at the electrode surface [46]. Meanwhile, the linear tail in the low-frequency region is associated with ion diffusion within the electrode structure. A clear distinction in impedance response is observed among the electrodes with different PEG concentrations. Notably, the NiCoS-P2 electrode exhibits the smallest semicircle diameter, indicating the lowest charge transfer resistance and, consequently, the most efficient electron transfer at the electrode–electrolyte interface. This behavior signifies accelerated redox kinetics and improved interfacial charge exchange. The extracted ESR values further support this observation, with the NiCoS-P2 electrode displaying the lowest resistance of 0.5 Ω (Table 1), indicative of superior electrical conductivity and minimized internal losses.

The long-term electrochemical durability of the optimized NiCoS-P2 electrode was systematically evaluated through extended GCD cycling at a high current density of 70 mA/cm^2^ (Figure 8d). The NiCoS-P2 electrode demonstrates outstanding durability, retaining approximately 84.16% of its initial capacitance after 12,000 continuous cycles, accompanied by a high coulombic efficiency of ~93%, indicating highly reversible redox processes with minimal energy loss during repeated operation. The excellent capacitance retention clearly reflects the robust structural integrity and electrochemical stability of the PEG-engineered electrode. The superior durability can be primarily attributed to the controlled role of PEG during synthesis, which governs nucleation and growth kinetics to yield a uniformly distributed, interconnected flower-like nanosheet architecture. This hierarchical structure provides sufficient mechanical flexibility to effectively accommodate the repeated volumetric expansion and contraction associated with OH^-^ ion insertion and extraction during cycling. The slight reduction in capacitance observed after extended cycling for the electrode may be attributed to several factors, including gradual ion trapping within micro- or mesoporous regions, minor surface reconstruction induced by repeated redox reactions, or partial blockage of electroactive sites [42]. However, the limited magnitude of this decay indicates that the overall electrode framework remains structurally stable and electrochemically active. Importantly, the consistently high coulombic efficiency throughout the cycling process confirms excellent reversibility of the battery-type Faradaic reactions and negligible parasitic side reactions, further validating the stability of the electrode–electrolyte interface.

**Figure 9:**
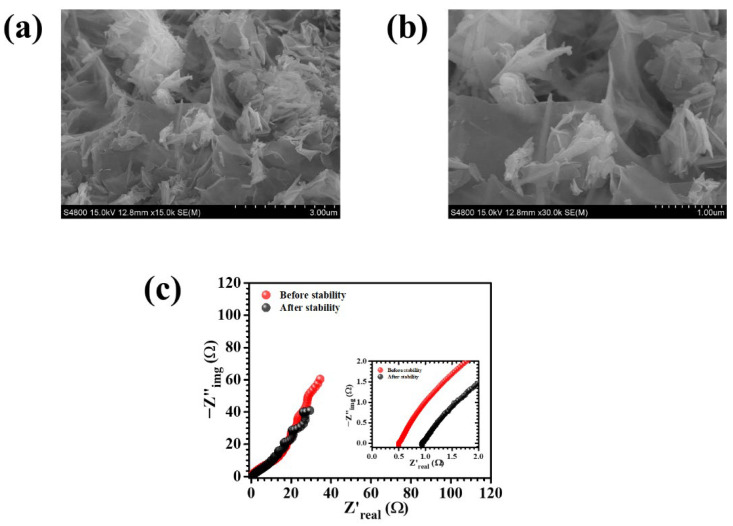
(**a**,**b**) FESEM images of the NiCoS-P2 electrode after cycling at different magnifications; (**c**) Nyquist plots before and after cycling showing changes in resistance.

To further substantiate the electrochemical stability and better understand the origin of capacitance decay, post-cycling analyses were carried out (Figure 9a,b). The FESEM images obtained after prolonged cycling show that the overall hierarchical nanosheet structure of the NiCoS-P2 electrode is largely retained, although slight structural distortion and localized aggregation can be observed. This suggests that the electrode maintains its structural integrity during repeated redox cycling, with minor changes likely resulting from mechanical stress and continuous ion insertion and extraction processes. In addition, the Nyquist plots recorded before and after cycling (Figure 9c) reveal a noticeable increase in charge transfer resistance, along with a slight change in the low-frequency region, indicating a gradual rise in internal resistance. Such behavior can be attributed to partial blockage of electroactive sites, ion trapping within the porous structure, and minor surface reconstruction during long-term operation. Despite these changes, the overall variation in impedance remains limited, and the morphology is largely preserved, confirming that the electrode experiences only minor degradation. Therefore, the observed capacitance decay can be mainly associated with slight kinetic limitations rather than significant structural failure, highlighting the robust nature of the PEG-assisted NiCoS-P2 electrode.

## 5. Electrochemical Performance of Asymmetric Supercapacitor Device

**Figure 10:**
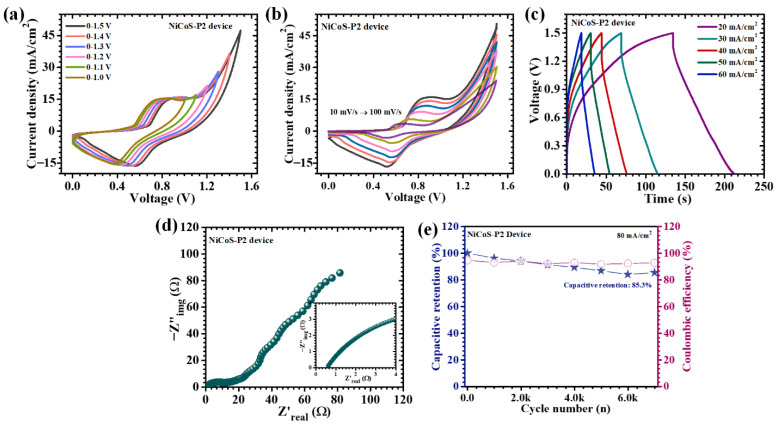
Electrochemical performance of the NiCoS-P2//AC asymmetric supercapacitor device: (**a**) CV curves at different voltage windows, (**b**) CV profiles at various scan rates, (**c**) GCD curves, (**d**) Nyquist plot, and (**e**) long-term cycling stability.

To evaluate the practical applicability of the optimized electrode beyond half-cell configurations, an asymmetric supercapacitor device (ASD) was assembled using the NiCoS-P2 electrode as the positive electrode and activated carbon (AC, Sigma-Aldrich U.S.) as the negative electrode. The selection of AC is based on its well-established electric double-layer capacitive (EDLC) behavior, high specific surface area, and excellent rate capability, which effectively complement the battery-type Faradaic characteristics of NiCo_2_S_4_. This rational combination enables a synergistic integration of surface-controlled and diffusion-controlled charge storage mechanisms, thereby maximizing the overall electrochemical performance of the device. Both electrodes were deposited onto nickel foam substrates to ensure low interfacial resistance, efficient current collection, and mechanical stability. The negative electrode was prepared by forming a homogeneous slurry comprising activated carbon, conductive carbon (acetylene black), and polyvinylidene fluoride (PVDF) binder dispersed in N-methyl-2-pyrrolidone (NMP). The slurry was uniformly coated onto nickel foam and dried under controlled conditions to achieve strong adhesion and structural integrity. The mass ratio of activated carbon, acetylene black, and PVDF was maintained at 80:10:10. The typical mass loading of the active material on the negative electrode was 2 mg cm^−2^. The coating was carried out using a doctor blade technique to ensure uniform film formation. A porous cellulose-based separator (filter paper) was employed, and the device was assembled in a two-electrode configuration with an effective electrode area of 2 × 3 cm^2^. The assembled device employed an aqueous alkaline electrolyte (2 M KOH) along with a porous separator to enable efficient ionic conduction while preventing electrical short-circuiting. The electrochemical behavior of the assembled NiCoS-P2//AC device was systematically investigated using CV, GCD, and EIS. To determine the optimal operating voltage window, CV measurement was conducted over progressively expanded potential ranges. The device maintained stable electrochemical behavior as the voltage window was increased stepwise, ultimately achieving a maximum stable operating range of up to ~1.5 V without noticeable distortion or abrupt current rise (Figure 10a). Further CV analysis at scan rates ranging from 10 to 100 mV/s reveals quasi-rectangular profiles embedded with distinct redox humps, shown in Figure 10b. This hybrid shape confirms the coexistence of EDLC behavior from the AC electrode and battery-type Faradaic reactions from the NiCo_2_S_4_ electrode. The retention of curve shape at higher scan rates indicates good electrochemical reversibility and efficient charge propagation within the device. The GCD profiles of the assembled device measured at different current densities (20 to 60 mA/cm^2^; Figure 10c) exhibit non-linear charge–discharge characteristics with evident voltage plateaus, confirming the dominant contribution of battery-type Faradaic processes from the positive electrode. The asymmetry in the charge–discharge curves further reflect the hybrid charge storage mechanism. At a current density of 10 mA/cm^2^, the device delivers an areal capacitance of 0.409 F/cm^2^, accompanied by an energy density of 0.128 mWh/cm^2^ and a power density of 2.99 mW/cm^2^ (Table 3). These values indicate a well-balanced energy–power relationship, which is essential for practical energy storage systems. Even at higher current densities, the device retains a significant fraction of its initial capacitance, demonstrating excellent rate capability. This performance can be attributed to the optimized electrode architecture, which enables rapid ion diffusion and efficient electron transport across the electrode–electrolyte interface.

EIS measurements provide further insight into the internal resistance and charge transfer behavior of the assembled device. The Nyquist plot (Figure 10d) exhibits a small semicircle in the high-frequency region followed by a nearly linear response at low frequencies, indicating efficient charge transfer kinetics and favorable ion diffusion behavior. The ESR of the device is determined to be approximately 0.62 Ω, reflecting low internal resistance and efficient ionic/electronic conduction pathways. The long-term cycling stability of the NiCoS-P2//AC device was evaluated over 7000 continuous charge–discharge cycles at a high current density of 80 mA/cm^2^ (Figure 10e). The device retains approximately 85.3% of its initial capacitance, along with a high coulombic efficiency of ~92%, demonstrating excellent electrochemical reversibility and durability. The outstanding cycling performance resulted from the synergistic structural and compositional features of the electrode materials. The PEG-assisted NiCo_2_S_4_ electrode provides a mechanically robust and hierarchical nanosheet framework that effectively accommodates volumetric changes during repeated redox cycling. Simultaneously, the AC electrode maintains stable EDLC behavior without structural degradation. Overall, the NiCoS-P2//AC asymmetric supercapacitor demonstrates a compelling combination of extended operating voltage, high capacitance, favorable energy–power output, and excellent cycling stability. These results clearly establish that PEG-assisted structural engineering of NiCo_2_S_4_ not only enhances half-cell electrochemical properties but also translates effectively into superior device-level performance.

## 6. Conclusions

In this work, NiCoS nanostructured electrodes were prepared using a PEG-assisted hydrothermal method to control their morphology. Among the synthesized samples, the NiCoS-P2 electrode showed a clear hierarchical nanosheet structure, which resulted in improved electrochemical performance. It delivered a high areal capacitance of 13.689 F/cm^2^ (specific capacitance of 6845 F/g) at 5 mA/ cm^2^ and maintained about 84.16% of its capacitance after 12,000 cycles. The assembled asymmetric supercapacitor device also showed good performance, with an areal capacitance of 0.409 F/cm^2^ (specific capacitance of 204.5 F/g), an energy density of 0.128 mWh/cm^2^, and a power density of 2.99 mW/cm^2^, along with 85.3% retention after 7000 cycles. These results are mainly due to the improved structure, which helps improve ion movement and effective use of active material. Overall, this approach offers a simple and reliable way to develop efficient NiCoS electrodes for practical energy storage applications.

## Figures and Tables

**Table 1:** Summary of kinetic and electrochemical parameters, including diffusion coefficients, b-values, and ESR of the investigated electrodes.

Sample Code	Diffusion Coefficient (cm^2^/s) × 10^−8^		b-Value	ESR (Ω)
	Oxidation	Reduction		
**NiCoS**	3.147	2.837	0.44	1.07
**NiCoS-P1**	8.927	8.938	0.47	0.62
**NiCoS-P2**	18.656	12.682	0.51	0.5
**NiCoS-P3**	5.92	4.905	0.45	0.85

**Table 2:** Electrochemical performance evaluation of the electrodes, presenting areal capacitance, areal capacity, specific capacitance, energy density, and power density at different current densities.

Sample Code	I (mA/cm^2^)	Areal Capacitance C_A_ (F/cm^2^)	Specific Capacitance C_G_ (F/g)	Capacity (mAh/cm^2^)	Energy Density ED (mWh/cm^2^)	Power Density PD (mW/cm^2^)
**NiCoS**	5	6.778	3389	0.282	0.085	0.57
	8	6.756	3378	0.281	0.084	0.92
	10	5.333	2667	0.222	0.067	1.20
	20	3.003	1502	0.109	0.04	3.28
	30	1.533	767	0.064	0.019	3.45
**NiCoS-P1**	5	12.978	6489	0.541	0.162	0.55
	8	12.667	6334	0.528	0.158	0.88
	10	6.222	3111	0.259	0.078	1.10
	20	4.444	2222	0.185	0.056	1.92
	30	2.156	1078	0.105	0.031	2.59
**NiCoS-P2**	5	13.689	6845	0.570	0.171	0.56
	8	13.111	6556	0.546	0.164	0.89
	10	11.778	5889	0.491	0.147	1.10
	20	7.556	3778	0.315	0.094	2.17
	30	4.667	2334	0.194	0.058	3.00
**NiCoS-P3**	5	9.778	4889	0.407	0.122	0.58
	8	9.556	4778	0.398	0.119	0.93
	10	5.556	2778	0.231	0.069	1.13
	20	3.333	1667	0.139	0.042	2.16
	30	2.036	1018	0.087	0.028	3.00

**Table 3:** Energy storage performance metrics of the NiCoS-P2//AC asymmetric supercapacitor device.

Sample Code	I (mA)	Areal Capacitance C_A_ (F/cm^2^)	Specific Capacitance C_G_ (F/g)	Capacity (mAh/cm^2^)	Energy Density ED (mWh/cm^2^)	Power Density PD (mW/cm^2^)
**NiCoS-P2//AC** **device**	20	0.409	204.5	0.085	0.128	2.99
	30	0.360	180.0	0.075	0.113	4.40
	40	0.320	160.0	0.067	0.100	5.63
	50	0.289	144.5	0.060	0.090	6.77
	60	0.213	106.5	0.044	0.067	7.06

## Data Availability

The original contributions presented in this study are included in the article. Further inquiries can be directed to the corresponding author.
